# Age of Acquisition Modulates Alpha Power During Bilingual Speech Comprehension in Noise

**DOI:** 10.3389/fpsyg.2022.865857

**Published:** 2022-04-25

**Authors:** Angela M. Grant, Shanna Kousaie, Kristina Coulter, Annie C. Gilbert, Shari R. Baum, Vincent Gracco, Debra Titone, Denise Klein, Natalie A. Phillips

**Affiliations:** ^1^Department of Psychology, Centre for Research in Human Development, Concordia University, Montreal, QC, Canada; ^2^Centre for Research on Brain, Language and Music, McGill University, Montreal, QC, Canada; ^3^School of Psychology, University of Ottawa, Ottawa, ON, Canada; ^4^Cognitive Neuroscience Unit, Montreal Neurological Institute, McGill University, Montreal, QC, Canada; ^5^School of Communication Sciences and Disorders, McGill University, Montreal, QC, Canada; ^6^Haskins Laboratories, New Haven, CT, United States; ^7^Department of Psychology, McGill University Montreal, Montreal, QC, Canada; ^8^Department of Neurology and Neurosurgery, Montreal Neurological Institute, McGill University, Montreal, QC, Canada; ^9^Bloomfield Centre for Research in Aging, Lady Davis Institute for Medical Research and Jewish General Hospital, McGill University Memory Clinic, Jewish General Hospital, Montreal, QC, Canada

**Keywords:** electrophysiology, alpha power, bilingualism, speech-in-noise, age of acquisition

## Abstract

Research on bilingualism has grown exponentially in recent years. However, the comprehension of speech in noise, given the ubiquity of both bilingualism and noisy environments, has seen only limited focus. Electroencephalogram (EEG) studies in monolinguals show an increase in alpha power when listening to speech in noise, which, in the theoretical context where alpha power indexes attentional control, is thought to reflect an increase in attentional demands. In the current study, English/French bilinguals with similar second language (L2) proficiency and who varied in terms of age of L2 acquisition (AoA) from 0 (simultaneous bilinguals) to 15 years completed a speech perception in noise task. Participants were required to identify the final word of high and low semantically constrained auditory sentences such as “Stir your coffee with a *spoon*” vs. “Bob could have known about the *spoon*” in both of their languages and in both noise (multi-talker babble) and quiet during electrophysiological recording. We examined the effects of language, AoA, semantic constraint, and listening condition on participants’ induced alpha power during speech comprehension. Our results show an increase in alpha power when participants were listening in their L2, suggesting that listening in an L2 requires additional attentional control compared to the first language, particularly early in processing during word identification. Additionally, despite similar proficiency across participants, our results suggest that under difficult processing demands, AoA modulates the amount of attention required to process the second language.

## Introduction

Listening to speech in noise is a part of everyday speech processing. Whether it’s the traffic outside or a conversation occurring in another room, speech partners are often engaged in not only the basics of speech production and comprehension, but the dual task of ignoring a non-target stimulus. The challenge of processing speech in noisy environments is further complicated in bilingual individuals who are required to listen and comprehend in both a native (L1) and a second (L2) language. Although research has examined speech-in-noise processing in monolinguals, there has been little focus on bilinguals despite estimates that more than half of the world’s population speaks more than one language (e.g., Grosjean, 2008). In the current study, we use electrophysiological measures to examine the recruitment of attentional resources during speech-in-noise processing in an L1 and an L2 in a well-controlled sample of bilingual participants. Additionally, we examine the potential role of the timing of L2 learning (i.e., age of acquisition; AoA) on speech-in-noise processing in L2.

Behavioral studies in monolinguals have shown that listening to speech in noise decreases comprehension accuracy (Kalikow et al., 1977; Bilger et al., 1984) and increases listener effort (Zekveld et al., 2014; McMahon et al., 2016; Pichora-Fuller et al., 2016). These effects are thought to be due in part to increased demands on working memory and selective attention systems compared to listening in quiet (for a review, see Wilsch and Obleser, 2016). However, the effects of speech degradation can be reduced if the context is semantically constrained. Behaviorally, a constraining sentence context (Davis et al., 2011) or a semantically related prime (Bernstein et al., 1989; Golestani et al., 2009) have been found to improve the accuracy of target word perception when processing speech in noise.

Electrophysiologically, studies have examined speech perception in noise using the N400 event-related potential (e.g., Connolly et al., 1992; Aydelott et al., 2006; Obleser and Kotz, 2011; Strauß et al., 2013; Carey et al., 2014; Coulter et al., 2020), a negative-going component that peaks approximately 400 ms following an eliciting stimulus. The N400 is elicited by semantic stimuli and its amplitude is inversely related to the semantic expectancy of the stimulus, such that it is larger when a target is semantically unexpected compared to when it is semantically expected (Kutas and Hillyard, 1980; Kutas and Van Petten, 1994; Kutas, 1997). Studies have found that N400 amplitude and the N400 effect (the difference in amplitude between unexpected and expected conditions) are attenuated, and the latency of the N400 is delayed in noise compared to quiet (e.g., Connolly et al., 1992; Aydelott et al., 2006; Obleser and Kotz, 2011; Strauß et al., 2013; Carey et al., 2014), suggesting that despite the beneficial effect of semantic constraint on behavioral performance, a processing cost remains.

Another method for examining electrophysiological measures is to decompose the waveform into its component frequency bands and compute the power in each of the frequency bands (i.e., time-frequency analysis), which have distinct functional correlates. Relevant to the current study, previous research has identified alpha oscillations (∼8–13 Hz) as a neural signature of cognitive effort, with an increase in power in the alpha band associated with increased cognitive effort and inhibition (e.g., Klimesch et al., 2007; Jensen and Mazaheri, 2010). Previous electro- and magneto-encephalography studies of speech-in-noise processing have used alpha power as a measure of attentional processes during speech-in-noise processing. Increases in alpha band power have been associated with increases in speech degradation (e.g., Obleser et al., 2012; Becker et al., 2013) and increased demands on attentional systems and inhibitory control (see Jensen and Mazaheri, 2010; Foxe and Snyder, 2011; for review).

When auditory degradation has been combined with manipulations of working memory, the increase in alpha power is super-additive (Obleser et al., 2012). More recently, Wostmann et al. (2017) manipulated the effort required for speech comprehension by increasing the acoustic detail in to-be-ignored distractor information and concluded that alpha power is related to top-down attentional control, with greater alpha power being positively associated with the effort required for speech comprehension rather than with acoustic degradation, *per se*. Additionally, studies that presented speech in quiet but manipulated the semantic constraint of the sentence find a decrease in alpha band power with increases in semantic constraint, which is thought to indicate the use of predictive mechanisms in sentence comprehension (Rommers et al., 2017; Wang et al., 2018).

### Bilingual Speech-in-Noise Comprehension

The current study builds on the existing literature by examining alpha power in L2 speech processing in noise. Previous literature on speech-in-noise processing has found L2 comprehension to be particularly sensitive to effects of noise (e.g., Mayo et al., 1997; Shi, 2010; Hervais-Adelman et al., 2014; Krizman et al., 2017). That is, the presentation of noise impairs speech comprehension in the L2 to a greater extent than in the L1. Furthermore, the limited literature suggests that L2 listeners may not be able to utilize semantic constraint under noisy conditions in the same way as in the L1. In one study, Hervais-Adelman et al. (2014) found that bilinguals only benefited from contextual information when processing speech-in-noise in their native language. More recently, Krizman et al. (2017) showed that bilinguals performed worse when perceiving sentences in noise in their L2 compared to monolinguals, whereas bilinguals performed better than monolinguals at perceiving tones in noise, suggesting that effects of acoustic degradation on L2 speech comprehension are dependent on linguistic knowledge. In contrast, other research has shown that bilinguals may benefit from a contextually supportive sentence context to a greater extent in their L2 compared to their L1 when listening to speech-in-noise (Chauvin and Phillips, in press).

Earlier research has further suggested that the effect of noise on L2 speech comprehension is moderated by L2 AoA, such that bilinguals with earlier ages of acquisition show smaller effects of noise (Mayo et al., 1997; Shi, 2010). More recently, Kousaie et al. (2019) observed that simultaneous bilinguals and those with an L2 AoA before age 5 show a benefit of contextual information in their L2 in terms of behavioral performance, whereas bilinguals who learned their L2 after age 5 did not. In addition to behavioral measures, Kousaie et al. (2019) examined neural responses during L1 and L2 speech processing in noise using functional magnetic resonance imaging, and observed that the absence of a behavioral benefit of context in the late bilinguals was accompanied by differences in neural recruitment of the inferior frontal gyrus in that group compared to simultaneous and early bilinguals. Additionally, using ERPs in a similar paradigm as Kousaie et al., Coulter et al. (2020) showed that bilinguals with different AoAs benefited from contextual information when processing sentences in their L2 in noise; however, ERP topography suggested that additional neural resources were recruited in sequential compared to simultaneous bilinguals. A common weakness in the studies that examine AoA is that AoA and proficiency are often confounded given that bilingual participants with later AoAs tend to be less proficient, although in both Kousaie et al. and Coulter et al. participant groups did not differ in terms of L2 proficiency. However, it remains unclear if the previously observed effects of AoA on L2 speech-in-noise processing are due to differences in AoA or proficiency. In fact, other behavioral research has shown that the ability to inhibit interference in a sentence interpretation task was positively correlated with L2 proficiency (Filippi et al., 2012); however, the paradigm used by Filippi et al. used was different than that used in the studies discussed above. More recently, others have also demonstrated a behavioral advantage in sentence recognition in noise in the L1 of bilinguals compared to monolinguals (Ferreira et al., 2019), while bilinguals in their L2 have been found to perform worse than monolinguals when the stimuli included a combination of words and sentences (Bsharat-Maalouf and Karawani, 2022). Importantly, Bsharat-Maalouf and Karawani (2022) also recorded electrophysiological responses to vowel sounds and found earlier auditory brain stem responses in noise in bilinguals compared to monolinguals, suggesting a different pattern of language group differences at the level of neural responses.

### Current Study

The current study compares the performance of highly proficient bilinguals who differ only in L2 AoA to control for the potential confounding effect of L2 proficiency. Furthermore, by evaluating alpha power during both L1 and L2 comprehension, we investigate whether domain general attentional control accounts for differences between L1 and L2 speech processing.

#### Hypotheses

Based on the current literature, we expected to observe differences in alpha power as a function of the following factors: Listening Condition (Quiet vs. Noise), Language (L1 vs. L2), Semantic Constraint (High vs. Low) and AoA (continuous). Specifically, we expected to observe:

1. Increased alpha power during speech comprehension in Noise compared to Quiet conditions.
2. Increased alpha power during L2 compared to L1 speech comprehension.
3. Increased alpha power for Low compared to High Constraint sentences.
4. A positive association between alpha power during L2 speech-in-noise comprehension and AoA, if AoA has an impact on speech processing in noise in L2.

## Materials and Methods

### Participants

Participants were 49 English/French bilinguals recruited from the Montréal community (mean age = 24.29 years, *SD* = 4.18; 36 females); 16 of these participants previously completed a similar speech perception in noise task during functional magnetic resonance imaging (see Kousaie et al., 2019 for details). Twenty-four participants identified English as their first language, and 25 identified French. Of the total sample, 14 participants were simultaneous bilinguals (i.e., learned both languages from birth), 6 of whom identified English as their dominant language, and 8 of whom identified French as their dominant language; See Table 1 for a summary of participant characteristics. All participants were right-handed with normal bilateral pure-tone hearing thresholds (i.e., <25 dB at 500, 1000, 2000, and 4000 Hz). Participants gave informed consent and received monetary compensation for participating.

**TABLE 1:** Summary of demographic, language, and cognitive task data, *n* = 49 (unless otherwise indicated), 36 females.

	Mean (*SD*)
Age	24.29 (*4.18*)
Education	15.32 (*1.73*)
Age of L2 acquisition^a^	4.27 (*3.63*)
L1 letter fluency^a^	36.65 (*9.71*)
L1 category fluency^b^	19.21 (*6.15*)
L2 letter fluency^a^	29.46 (*9.28*)
L2 category fluency^a^	16.00 (*5.42*)
L1 coefficient of variation^a^	0.37 (*0.20*)
L2 coefficient of variation^a^	0.40 (*0.22*)
L1 self-reported speaking proficiency	6.86 (*0.41*)
L1 self-reported listening proficiency	6.94 (*0.32*)
L2 self-reported speaking proficiency	5.79 (*1.03*)
L2 self-reported listening proficiency	6.26 (*0.87*)
L1 percentage of language use^c^	58.63 (*25.83*)
L2 percentage of language use^c^	41.14 (*25.75*)
Digit span forward^b^	7.04 (*1.22*)
Digit span backward^b^	5.15 (*1.32*)
Digit span sequencing^a^	6.13 (*1.14*)
Letter-number sequencing^a^	5.69 (*1.13*)
Matrix reasoning^a^	12.04 (*2.39*)

*^a^ Data are missing for one participant.*

*^b^ Data are missing for two participants.*

*^c^ Data are missing for five participants.*

### Materials

#### Speech Perception in Noise Task

The current study used the same speech perception in noise task as Coulter et al. (2020). A total of 240 sentences were adapted from the Revised Speech Perception in Noise Test (SPIN-R; Kalikow et al., 1977). The final words of the SPIN-R stimuli were of high or low predictability based on the amount of semantic context in the sentence (i.e., high- vs. low-constraint, e.g., “The lion gave an angry roar.” vs. “He is thinking about the roar.”; see Kalikow et al., 1977 for details on sentence creation). Sixty high-constraint and sixty low-constraint English sentences were selected from the eight original lists of the SPIN-R test. The selected high and low constraint English sentences were matched on both number of words (high-constraint: *M* = 5.5, *SD* = *0.81*; low-constraint: *M* = 4.9, *SD* = *0.79*) and number of syllables (high-constraint: *M* = 6.5, *SD* = *0.70*; low-constraint: *M* = 6.6, *SD* = *0.70*).

An additional 120 SPIN-R sentences (60 high-constraint and 60 low-constraint) were selected and adapted to French. To match high and low constraint French sentences on sentence length, some French sentences were slightly modified translations of original SPIN-R sentences, e.g., “The bread was made from whole wheat” was adapted to “Le pain brun est fait de blé.” French sentences were distinct from the English sentences used in this experiment. High and low constraint French sentences were also matched on number of words (high-constraint: *M* = 5.8, *SD* = 1.01; low-constraint: *M* = 5.0, *SD* = 1.15) and number of syllables (high-constraint: *M* = 7.7, *SD* = 1.04; low-constraint: *M* = 7.3, *SD* = 1.21). Target terminal French words were either monosyllabic or disyllabic; disyllabic terminal words were included to accommodate the other stimulus inclusion criteria. English and French terminal words were also matched on spoken frequency (English: *M* = 20.5, *SD* = 27.50; French: *M* = 24.4, *SD* = 28.90), phonological neighborhood density (English: *M* = 15.4, *SD* = 9.22; French: *M* = 16.4, *SD* = 7.38), imageability (English: *M* = 539.5, *SD* = 65.77; French: *M* = 563.0, *SD* = 48.44), and familiarity (English: *M* = 524.5, *SD* = 51.36; French: *M* = 517.4, *SD* = 55.09) using the MRC Psycholinguistic Database (Coltheart, 1981), Lexique 3 (New et al., 2001; New, 2006), and the Corpus of Contemporary American English (Davies, 2008).

All sentences were recorded by a female, simultaneous bilingual speaker of Canadian English and French. Sentences were recorded in a sound-attenuated booth using an Olympus recorder with a 44.1 kHz sample-rate and 32-bit resolution. Sentence stimuli were presented to participants in both a quiet condition and a noise condition. The background noise consisted of multi-talker babble adapted from Bilger et al. (1984) such that the original eight-talker babble was overlaid three times with a slight temporal jitter to create a babble mask that was less variable in its intensity fluctuations (Winneke and Phillips, 2011).

There were eight experimental conditions (four conditions in each language) in our 2 × 2 × 2 design: High-constraint sentences in quiet, low-constraint sentences in quiet, high-constraint sentences in noise, and low-constraint sentences in noise were presented in each language. Within each language, each target word was presented in all four conditions, but stimuli were divided into two lists so that each target word was heard only twice in each list by any given participant. For example, the terminal word “*spoon*” was heard in the high-constraint quiet and the low-constraint noise conditions in List 1 and was heard in the low-constraint quiet and high-constraint noise conditions in List 2. Each list consisted of eight experimental blocks, as described above. Lists were blocked by listening condition (quiet and noise) and language (English and French), which were counterbalanced within each list. Low constraint and high constraint sentences were pseudo-randomly intermixed within each block such that there were an equal number of each but no more than three consecutive sentences of the same type. Each participant heard only one list and lists were counterbalanced across participants.

#### Language Proficiency Measures

Participants completed a language history questionnaire and letter and category verbal fluency tasks, and animacy judgment tasks in each of their languages; see Table 1. Additional language tasks not discussed here included a story reading and comprehension task, picture description, and sentence repetition.

Participants self-rated their proficiency in speaking and understanding both of their languages on a scale from 1 to 7 (1 being not at all proficient and 7 being native-like proficiency). All participants rated themselves as being highly proficient in their L2. Speaking and listening proficiencies ranged from 5 to 7 for L1 and from 4 to 7 for L2. Participants varied in the percentage of their total conversations in which they used each of their languages, with the percentage of L2 use ranging from 5 to 95% of all conversations.

In the fluency tasks, participants were asked to say as many words as they could (excluding proper nouns, numbers, and words that differed only in their suffix) that began with a given letter of the alphabet or that fit with a given category in 1 min. The letters included *F*, *A*, and *S* for the English letter fluency and the letters *P*, *F*, and *L* for the French letter fluency. The number of words produced for all three letters, within each language, were summed to give a single letter fluency score for each language. For category fluency, the categories were *animals* and *fruit* for English and French, respectively.

For the animacy judgment task, participants judged whether a presented word was living (“m,” right key press) or non-living (“z,” left key press) as quickly and accurately as possible (Segalowitz and Segalowitz, 1993). During the task, each word was presented in white 18-point Courier New font on a black background using E-Prime 2.0 software on a Dell Precision M2800 15” laptop running Windows 7 professional. Trials ended when the participant responded, and there was a 250-ms interstimulus interval. Participants first completed a neutral block, where they had to judge if the stimulus was a letter or a number. After the neutral block, participants completed separate blocks of the task in each language. Each block began with eight practice trials, followed by 64 unique nouns. The French words were not translations of the English words, and blocks were matched for the number of animate and inanimate judgments. Data from the animacy judgment task were used to calculate the coefficient of variation, a measure of automaticity in language processing (Segalowitz and Segalowitz, 1993) that has previously been taken as an objective measure of relative L2 proficiency (e.g., Segalowitz and Frenkiel-Fishman, 2005; Kousaie and Phillips, 2012, 2017).

#### Measures of Cognitive Ability

Participants completed several subtests of the Weschler Adult Intelligence Scale, Fourth edition (WAIS-IV; Wechsler, 2008) to ensure that cognitive functioning was within the normal range. Participants completed the Digit Span (forward, backward, and sequencing), Letter-Number Sequencing, and Matrix Reasoning subtests; see Table 1 for scaled scores. For the digit span tasks, participants were read a series of digits by the experimenter and were asked to repeat the digits in the same order as they were presented (i.e., forward), in the backward order (i.e., backward) or in ascending order (i.e., sequencing). The number of digits started at two and increased by one digit to a maximum of nine for the forward and sequencing subtests, and a maximum of eight for the backward subtest. The task ended when the participant got both trials of a span length incorrect.

In the Letter-Number Sequencing task, participants were presented with a series of numbers and letters and were asked to repeat the numbers first in ascending order, followed by the letters in alphabetical order. The series started with one number and one letter and increased by one item up to a maximum of eight items. The task ended when the participant got all three trials of a span length incorrect.

For the Matrix Reasoning subtest, participants were presented with a series of 26 designs increasing in complexity and were required to identify patterns in each design by selecting the item that completed the pattern from five alternatives. The task ended when the participant obtained three consecutive incorrect responses.

### Procedure

Participants completed two testing sessions on two different days. In the first session, participants completed the pure-tone hearing and language proficiency assessments, as well as several executive function tests that will not be further reported here. In addition, the participants completed a language background questionnaire in which they self-reported detailed information regarding their L1 and L2 language proficiency, AoA, and patterns of language use. In the second session, participants performed the experimental speech perception in noise task, while their electroencephalogram was recorded. Following the experimental task, participants completed three other tasks that are not reported here (see Giroud et al., 2020; Gilbert et al., 2021). For all tasks, participants were seated in a sound attenuated booth in front of a computer monitor. Participants first completed a practice block of the speech perception in noise task in English and French. Practice trials consisted of 41 sentences (22 English and 19 French), half high-constraint and half low-constraint sentences. Five sentences in each language were presented in quiet and the rest in noise. Participants then completed one list (i.e., 240 sentences) of the experimental task. Sentences were binaurally presented through EARLINK tube ear inserts (Neuroscan, El Paso, TX, United States) using Inquisit 4.0 (Millisecond Software, Washington). In the noise condition, stimuli were presented at a signal-to-noise ratio of + 1 dB as this gave a 30% error rate in the most challenging condition (i.e., low-constraint L2 sentences presented in noise) during pilot testing. During sentence presentation, a fixation cross was presented on the computer screen. After each sentence was presented, participants were prompted to repeat the final word of the preceding sentence 1,000 ms after the end of the sentence (i.e., when “Final Word?” appeared on the computer screen). Responses were manually scored as correct or incorrect by the experimenter. In addition to verbatim correct responses, responses were accepted as correct if the participant made a pluralization error that was semantically and syntactically correct within the context of the sentence or if participants included the determiner associated with the target word in the French sentences. Only correct trials were included in EEG analyses.

### Electroencephalogram Data Acquisition and Analysis

Electrophysiological activity was recorded from a 64 Ag-AgCl active electrodes using the international 10/20 system of electrode placement (Biosemi, Amsterdam, NL) with a sampling rate of 2048 Hz. Additional facial electrodes were placed above and below the left eye and on the left and right canthi to record horizontal and vertical eye movements.

Processing of EEG data was conducted using BrainVision Analyzer 2.0.3 (Brain Products, Gilching, DE). Data were screened manually to remove visible artifacts and sections of the recording in between experimental blocks. All scalp electrodes were re-referenced offline to the average of electrodes placed on the left and right earlobes. A low-pass filter of 100 Hz and a high-pass filter of 0.01 Hz were applied, as well as a DC drift correction. Artifacts from vertical and horizontal eye movements were removed using the Ocular Correction Independent Components Analysis. Following ocular correction, the data were segmented into 1,500 ms intervals, with a 500 ms pre-stimulus baseline period before the onset of the sentence final word, and a 1,000 ms post-stimulus interval. Artifact rejection was semi-automatic, and segments were removed from the analysis if the absolute difference between two adjacent data points within a segment exceeded 50 microvolts, if the difference between the maximum and minimum amplitude within a segment exceeded 200 microvolts, or if the activity within a segment fell below 0.5 microvolts. An average of 26% of trials was removed for each participant. Following artifact rejection, each condition was segmented and baseline-corrected individually. Only correct trials were included; thus, a greater number of trials was excluded on average in the noise, low constraint, and L2 conditions, with the minimum number of trials in the L2 Low Constraint Noise condition (mean = 19, or 65% of trials). To obtain time-frequency representations of the data, we applied a Morlet transformation to the data between 5 and 40 Hz (35 steps), with a cycle parameter of 5. For each condition we then subtracted the evoked power from the total power of the transformed data to measure induced power. Time-frequency data from 7.5–12 Hz were exported for statistical analysis in 100 ms time windows from 100 to 700 ms post stimulus.

### Statistical Analysis of the Time-Frequency Data

Statistical analyses of the induced time-frequency data consisted of a linear mixed-effects model with random effects for subjects using the lme4 package (version 1.1–19) of R (version 3.5.1). Based on the typical distribution of the auditory N400 (Connolly et al., 1992; Connolly and Phillips, 1994; D’Arcy et al., 2004; van den Brink et al., 2006) and to reduce our familywise Type I error rate (Luck and Gaspelin, 2017), alpha power was operationalized as the average power in the 7.5–12 Hz frequency range at electrodes CPz and Pz.

The analysis included contrast-coded fixed effects for Language (L1 = −0.5, L2 = 0.5), Semantic Constraint (high = −0.5, low = 0.5) and Listening Condition (quiet = −0.5, noise = 0.5) in a 2 × 2 × 2 factorial design. Additional continuous fixed effects were estimated for AoA, time window, and Task Accuracy of repeating the final word (mean values per participant per condition). Although no predictions were made with respect to Time, given the precise temporal resolution afforded by EEG we included time as a factor to examine whether any of the effects of interest interacted with time. Time was scaled such that the time windows (100–200 ms, 200–300 ms, … 600–700 ms) were entered as values from 1 to 6. AoA and Time were allowed to interact with our other experimental factors listed above, whereas Task Accuracy was included as a separate fixed effect. Accuracy performance was standardized in the form of z-scores before inclusion in the model. Random effects included random intercepts for subjects. Random effects were limited to random intercepts per participant given that a) we estimated condition level averages as our dependent variable and b) the majority of our experimental factors have only two levels, which is not optimal for random slope estimation (Bolker, 2012). Models were fit using a restricted maximum likelihood estimation technique. A fixed effect was considered significant if the absolute value of the *t*-statistic was greater than or equal to 2.0 (Linck and Cunnings, 2015) and the *p*-values reported in Supplementary Table 1 were estimated using sjPlot’s tab_model function (version 2.6.1).

### Statistical Analysis of Behavioral Accuracy

Condition-level accuracy on the speech-in-noise task was evaluated in a linear mixed-effects model with random intercepts for subjects using the lme4 package (version 1.1–19) of R (version 3.5.1). Similar to the analysis of the electrophysiological data, the analysis included contrast-coded fixed effects for Language (L1 = −0.5, L2 = 0.5), Semantic Constraint (high = −0.5, low = 0.5) and Listening Condition (quiet = −0.5, noise = 0.5), as well as AoA as a continuous fixed effect. Fixed effects were evaluated using the same criteria and packages as in the time-frequency analysis.

## Results

### Verbal Fluency

Participants scored higher in L1 letter fluency (*M* = 36.65; *SD* = 9.71) compared to L2 letter fluency (*M* = 29.46; *SD* = 9.28; paired *t*(47) = 4.67, *p* < 0.001). Similarly, participants scored higher in L1 category fluency (*M* = 19.21; *SD* = 6.15) compared to L2 category fluency (*M* = 16.00; *SD* = 5.42; paired *t*(46) = 2.48, *p* = 0.02).

### Animacy Judgment

Participants’ reaction times (RTs) on the animacy judgment task were assessed in terms of the coefficient of variation, i.e., their standard deviation divided by their mean RT. As automatization in a language increases, the coefficient of variation decreases (Segalowitz and Segalowitz, 1993). The coefficient of variation in L1 (*M* = 0.37, *SD* = 0.20) was not significantly different from L2 (*M* = 0.40, *SD* = 0.22; *t*(47) = −1.14, *p* = 0.26), indicating a similar degree of automaticity across L1 and L2, despite greater verbal fluency in L1 compared to L2.

### Role of Age of L2 Acquisition

In our sample, average age of L2 acquisition was 4.27 years (*SD* = 3.63) and ranged from 0 to 15 years. We evaluated the influence of age of acquisition on participants’ proficiency by running a multivariate regression that evaluated the predictive power of AoA on L2 category fluency, L2 letter fluency, and the difference between the coefficient of variation in L2 and L1. Overall, the influence of AoA was not significant (*F*(3,45) = 1.62, *p* = 0.20), indicating that participants’ L2 proficiency was not confounded with AoA.

### Revised Speech Perception in Noise Test Behavioral Accuracy

**FIGURE 1:**
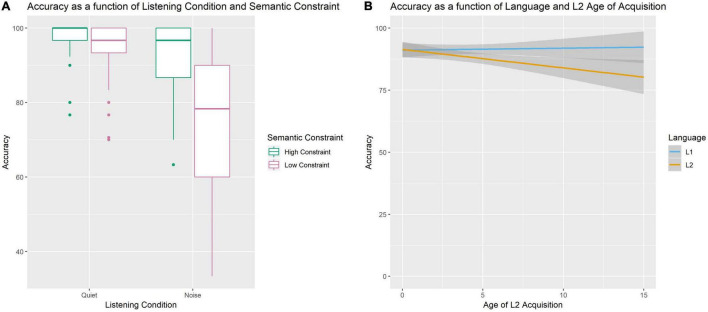
Accuracy Performance on the SPIN task. Panel **(A)** displays an interaction between Listening Condition and Semantic Constraint such that the effect of Semantic Constraint is larger in noisy conditions. Panel **(B)** displays an interaction between AoA and Language such that accuracy in the L2 decreases as L2 AoA increases.

All participants were more accurate on high constraint sentences compared to low constraint sentences (Beta Estimate = −10.55, CI [−13.54 −7.78], *p* < 0.001). Similarly, all participants were more accurate while perceiving speech in quiet compared to noise (Beta Estimate = −12.30, CI [−15.18 −9.42], *p* < 0.001). However, the decrease in accuracy for the noise compared to the quiet condition was greater for low than high constraint sentences (see Figure 1A; Beta Estimate = −16.50, CI [−22.26 −10.74], *p* < 0.001). Additionally, there was a Language by AoA interaction (see Figure 1B; Beta Estimate = −0.82, CI [−1.37 −0.28], *p* = 0.003) such that performance was overall less accurate in L2 compared to L1 for bilinguals with later ages of L2 acquisition.

### Analysis of Induced Alpha Power

**FIGURE 2:**
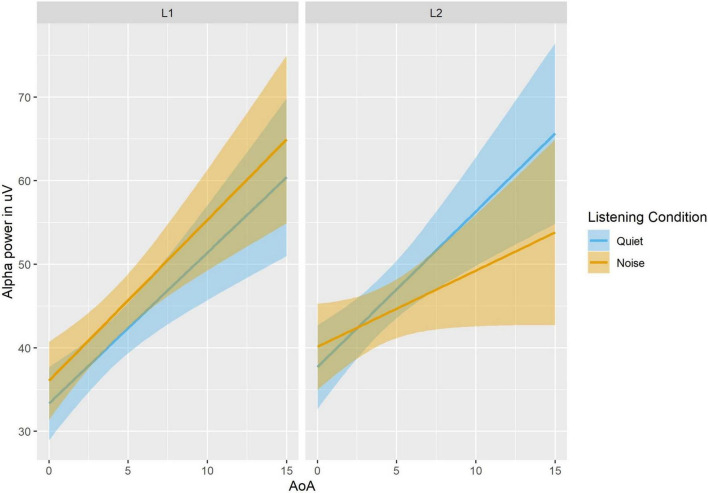
Alpha power as a function of Language, Listening Condition and AoA. In Quiet, alpha power is positively correlated with AoA, for each language, with overall higher alpha for L2. In Noise, alpha power is still positively correlated with AoA, but alpha power is lower in the L2 than the L1 for later AoA.

Results of the mixed-effect analyses are summarized in Supplementary Table 1 and depicted in Figure 2. Hypotheses 1 and 2 were supported by main effects of Language (Beta Estimate = 6.57, CI [2.08 11.07], *p* = 0.004; higher alpha power in L2 than L1), and Listening Condition (Beta Estimate = 4.66, CI [0.18 9.15], *p* = 0.042; higher alpha power in noise than quiet). The results did not support hypothesis 3 given that there was no significant main effect of Semantic Constraint (Beta Estimate = −3.67, CI [−8.16 0.81], *p* = 0.11), or interactions involving Semantic Constraint (all *p*s > 0.14). Additional main effects included: Time (Beta Estimate = −0.69, CI [−1.27 −0.12], *p* = 0.019; decreased alpha power over time) and Task Accuracy (Beta Estimate = 0.92, CI [0.11 1.74], *p* = 0.027; lower alpha was associated with lower accuracy). In terms of hypothesis 4, the main effect of Listening Condition was moderated by a two-way interaction with AoA (Beta Estimate = −0.98, CI [−1.82 −0.14], *p* = 0.022) and further by a three-way interaction between Listening Condition, Language, and AoA (Beta Estimate = −1.84, CI [−3.52 −0.16], *p* = 0.032), showing that (1) later AoA was associated with increased alpha in both L1 and L2 overall, (2) later AoA was associated with increased alpha in L2 compared to L1 only in quiet, and (3) later AoA was associated with increased alpha in quiet compared to noise in the L2.

## Discussion

Our study examined speech-in-noise processing in bilinguals who varied in their L2 AoA. Participants identified the terminal word of sentences that varied in terms of semantic constraint and we examined behavioral performance and alpha power as a measure of attentional control. We hypothesized that we would observe (1) increased alpha power during speech comprehension in the more difficult noise condition compared to quiet, (2) increased alpha power during L2 as compared to L1 speech comprehension, indicating more effortful processing, and (3) increased alpha power for low compared to high constraint sentences, and (4) that the increase in alpha power for L2 processing would positively correlate with L2 AoA. Our findings partially support these hypotheses.

The direction of the main effects of Listening Condition and Language supported hypotheses 1 and 2 – there was an increase in alpha power when processing speech in noise compared to quiet and in L2 compared to L1. Hypothesis 4 predicted an interaction between Listening Condition, Language, and AoA such that increasing AoA was expected to be associated with increased alpha power in noise in the L2. Although we observed a significant 3-way interaction, the source of the interaction did not support our hypothesis. In fact, later AoA was associated with increased alpha in L2 compared to L1 in quiet only, and there was a decrease in alpha power in L2 noise compared to L2 quiet. This pattern of results is distinct from the super-additive pattern we had hypothesized based on Obleser et al. (2012). However, our finding is consistent with activation patterns in the inferior frontal gyrus observed by Kousaie et al. (2019) using a similar task and group of participants. Like Kousaie et al., we interpret this finding as indicating that the observed pattern of decreased alpha power in L2 noise compared to L2 quiet may reflect resource exhaustion in the most challenging condition. This interpretation is also consistent with the observed interaction between Language and Listening Condition showing an increase in alpha for the noise compared to the quiet condition in L1 only, and the main effect of Language showing greater overall alpha power in L2 compared to L1, suggesting that both the noise and quiet conditions in L2 recruited similarly greater attentional resources than listening in L1. The behavioral results also show a decrease in accuracy for noise compared to quiet conditions that is larger in L2 than L1, providing additional evidence that this condition is more effortful.

In the case of alpha power, our interpretation of the absence of an increase in alpha power during the most difficult listening condition being the result of an exhaustion of available resources is consistent with several studies that fail to find increases in alpha power under incomprehensible/impossible task conditions (e.g., Becker et al., 2013; Wisniewski et al., 2017). Although our SPIN task was not impossible, as demonstrated by participants’ accuracy scores, it may be that the increase in alpha in response to task demands resembles a U-shaped function, wherein alpha power is low under easy and highly difficult conditions, and increases at medium processing loads. Given previous work that observed super additive effects of WM load and noise was conducted at the word level, it may be that the working memory tasks used in those studies never reached sufficient difficulty to observe a reduction in alpha power (Obleser et al., 2012; Wostmann et al., 2017). In contrast, our data are based on sentence-level processing in both a stronger and a weaker language, and consequently it is plausible that, particularly when L2 AoA is late, our task may have been sufficiently difficult to reach the point where additional alpha power was no longer beneficial. Further support for this interpretation comes from our behavioral results, which show decreases in performance in L2 with later AoA.

Although we demonstrated an association between alpha power and both noise and language in bilinguals, we did not observe an influence of semantic constraint on alpha power, thus not supporting hypothesis 3. This is inconsistent with previous studies that have found a decrease in alpha power for highly constraining sentences (e.g., Rommers et al., 2017; Wang et al., 2018), although these studies only examined processing in quiet. Despite the absence of an effect of constraint in the electrophysiological data, behaviorally we show that bilinguals benefit from semantic constraint in both languages, particularly in noise, and show improved behavioral performance in high constraint conditions. Our behavioral findings are consistent with the behavioral results from Coulter et al. (2020) with a partially overlapping sample of participants. However, Coulter et al. also showed an effect of semantic constraint on N400 amplitude, with larger amplitudes for low compared to high constraint sentences. In contrast, other previous work has found that bilinguals who learn their L2 after age 5 years do not benefit from semantic constraint in L2 noise (Kousaie et al., 2019); however, in that study the signal to noise ratio was lower than in the current study, thus further increasing the difficulty of speech processing and potentially attributing to the difference in findings. In the current study, we observe interactions with AoA, such that L2 speech-in-noise processing performance decreased at later AoAs (see Figure 1B), but these effects do not outweigh the benefits of semantic constraint on speech perception in noise in our highly proficient bilingual sample.

Further research will be needed to understand the mechanisms driving the effect of AoA during speech-in-noise processing, but one potential avenue for research could investigate the role of individual differences in phonetic perception in the L2, a skill that is known to be optimally sensitive during infancy (Werker et al., 1981). *Post hoc* correlations between the accuracy data on our SPIN task and participants’ frequency following response (i.e., an electrophysiological measure of the fidelity of neural encoding of sound) to vowels, a task that was completed later in the testing session (see Giroud et al., 2020 for details) revealed a positive relationship between these two measures. This supports the hypothesis that AoA may be related to more efficient lower-level phonetic processing leading to improved speech processing in difficult listening conditions. Given that we observed a greater alpha response at earlier time windows, and that participants with earlier AoAs show reduced alpha power compared to participants with later AoAs, our results are congruent with an interpretation that emphasizes the role of both language experience and bottom-up processing in speech perception in noise in an L2.

More broadly, our higher-order interactions with Language, Listening Condition, and AoA suggest that alpha reflects inhibitory processing during attentional control in bilingual auditory language processing, as has been previously demonstrated in vision (e.g., Engel et al., 2001). These data support hypotheses positing that bilinguals use domain-general cognitive control systems to manage the cognitive challenges associated with L2 language processing (e.g., Green, 1998; Green and Abutalebi, 2013). Furthermore, the participants in this study varied with respect to their L1, with approximately half of the participants reporting English to be their L1 and half reporting French as their L1, suggesting that the observed effects are relevant to bilingual language processing and not specific to a particular L2, at least in terms of the languages used here.

## Conclusion

The current data extend our understanding of alpha power to the bilingual context, showing that alpha power is sensitive to the attentional control demands associated with L2 speech comprehension, and that age of acquisition, beyond proficiency alone, predicts the degree of attentional control necessary for bilingual speech processing in noise. Future studies should build on our findings to examine, for example, whether experiential factors like AoA—which we have shown here to be associated with overall alpha power—are also associated with differences in the source of neural recruitment. These results represent an initial step toward broadening our understanding of naturalistic speech processing in ubiquitous conditions, such as in noisy environments and in a non-native language.

## Data Availability Statement

The dataset presented in this article is not readily available because participants did not provide consent for open access to their data. Requests to access the dataset should be directed to the corresponding author.

## Ethics Statement

The studies involving human participants were reviewed and approved by Institutional Review Board of the Faculty of Medicine and Health Sciences, McGill University. The patients/participants provided their written informed consent to participate in this study.

## Author Contributions

AMG and SK: formal analysis, data curation, writing (original draft and editing). ACG: formal analysis, writing (review and editing). KC: data curation, writing (review and editing). SB, VG, DT, and DK: conceptualization, writing (review and editing), funding acquisition. NP: conceptualization, writing (review and editing), funding acquisition, supervision. All authors contributed to the article and approved the submitted version.

## Conflict of Interest

The authors declare that the research was conducted in the absence of any commercial or financial relationships that could be construed as a potential conflict of interest.

## Publisher’s Note

All claims expressed in this article are solely those of the authors and do not necessarily represent those of their affiliated organizations, or those of the publisher, the editors and the reviewers. Any product that may be evaluated in this article, or claim that may be made by its manufacturer, is not guaranteed or endorsed by the publisher.
