# Supplementary material for: Age of Acquisition Modulates Alpha Power During Bilingual Speech Comprehension in Noise
Source: Front Psychol. 2022 Apr 25;13:865857. doi: 10.3389/fpsyg.2022.865857 (PMC9083356; doi:10.3389/fpsyg.2022.865857)
Supplement: Supplementary file 1 [file Data_Sheet_1.PDF]

Supplementary Table 1. *Summary of linear mixed-effects analysis.*

	Alpha power		
**Predictors**	*Estimates*	*CI*	*p*
(intercept)	39.12	23.49 – 54.75	**<0.001**
Task accuracy (*Z*-scored)	0.92	0.11 – 1.74	**0.027**
Language	6.57	2.08 – 11.07	**0.004**
Language * Semantic constraint	0.38	-8.60 – 9.35	0.934
Language * Semantic constraint * Listening condition	1.51	-16.43 – 19.45	0.869
Language * Semantic constraint * Listening condition * Time (scaled)	0.70	-3.91 – 5.31	0.766
Language * Semantic constraint * Listening condition * Time (scaled) * AoA	-0.64	-1.50 – 0.22	0.145
Language * Semantic constraint * Listening condition * AoA	2.04	-1.31 – 5.40	0.233
Language * Semantic constraint * Time (scaled)	-0.25	-2.55 – 2.05	0.831
Language * Semantic constraint * Time (scaled) * AoA	0.05	-0.38 – 0.48	0.817
Language * Semantic constraint * AoA	0.18	-1.50 – 1.86	0.834
Language * Listening condition	1.16	-7.81 – 10.13	0.801
Language * Listening condition * Time (scaled)	-0.39	-2.69 – 1.91	0.739
Language * Listening condition * Time (scaled) * AoA	0.21	-0.22 – 0.65	0.328
Language * Listening condition * AoA	-1.84	-3.52 – -0.16	**0.032**
Language * Time (scaled)	-0.78	-1.93 – 0.38	0.187
Language * Time (scaled) * AoA	0.01	-0.21 – 0.22	0.946
Language * AoA	-0.42	-1.26 – 0.43	0.334
Semantic constraint	-3.67	-8.16 – 0.81	0.109
Semantic constraint * Listening condition	-0.35	-9.32 – 8.62	0.939
Semantic constraint * Listening condition * Time (scaled)	-0.05	-2.36 – 2.25	0.964
Semantic constraint * Listening condition * Time (scaled) * AoA	0.09	-0.34 – 0.52	0.685
Semantic constraint * Listening condition * AoA	-0.47	-2.15 – 1.20	0.580
Semantic constraint * Time (scaled)	0.60	-0.55 – 1.75	0.305
Semantic constraint * Time (scaled) * AoA	-0.02	-0.24 – 0.19	0.834
Semantic constraint * AoA	0.63	-0.21 – 1.47	0.140
Listening condition	4.66	0.18 – 9.15	**0.042**
Listening condition *Time (scaled)	-0.59	-1.75 – 0.56	0.312
Listening condition *Time (scaled) * AoA	0.16	-0.06 – 0.37	0.150
Listening condition *AoA	-0.98	-1.82 – -0.14	**0.022**
Time (scaled)	-0.69	-1.27 – -0.12	**0.019**
Time (scaled) * AoA	0.04	-0.07 – 0.15	0.438
AoA	1.50	-1.42 – 4.42	0.320
**Random Effects**		Variance	
Participants		1354.75	
Residual		262.93	
**Model Fit**			
Marginal R2 / Conditional R2		0.026 / 0.842	
N participants		50	
Observations		2400	
